# Prognostic value of albumin-to-globulin ratio in COVID-19 patients: A systematic review and meta-analysis

**DOI:** 10.1016/j.heliyon.2022.e09457

**Published:** 2022-05-18

**Authors:** Juan R. Ulloque-Badaracco, Melany D. Mosquera-Rojas, Enrique A. Hernandez-Bustamante, Esteban A. Alarcón-Braga, Percy Herrera-Añazco, Vicente A. Benites-Zapata

**Affiliations:** a Escuela de Medicina, Universidad Peruana de Ciencias Aplicadas, Lima, Peru; b Sociedad Científica de Estudiantes de Medicina de la Universidad Peruana de Ciencias Aplicadas, Lima, Peru; c Sociedad Cientifica de Estudiantes de Medicina de la Universidad Nacional de Trujillo, Trujillo, Peru; d Grupo Peruano de Investigación Epidemiológica, Unidad para la Generación y Síntesis de Evidencias en Salud, Universidad San Ignacio de Loyola, Lima, Peru; e Universidad Privada San Juan Bautista, Lima, Peru; f Instituto de Evaluación de Tecnologías en Salud e Investigación – IETSI, EsSalud, Lima, Peru; g Unidad de Investigación para la Generación y Síntesis de Evidencias en Salud, Vicerrectorado de Investigación, Universidad San Ignacio de Loyola, Lima, Peru

**Keywords:** Albumin, COVID-19, Globulin, Prognosis

## Abstract

**Background and aims:**

The albumin-to-globulin ratio (AGR) has been used to predict severity and mortality in infectious diseases. The aim of this study is to evaluate the prognostic value of the AGR in COVID-19 patients.

**Methods:**

A systematic review and meta-analysis were conducted. We included observational studies assessing the association between the AGR values upon hospital admission and severity or all-cause mortality in COVID-19 patients. In the meta-analyses we used random effect models. Risk of bias was assessed using the Newcastle-Ottawa Scale (NOS). The effect measures were expressed as mean difference (MD) and their 95% confidence intervals (CI). We performed Egger's test and funnel plots to assess the publication bias.

**Results:**

The included studies had a total of 11356 patients corresponding to 31 cohort studies. Severe COVID-19 patients had lower AGR values than non-severe COVID-19 patients (mean difference (MD), −0.27; 95% IC, −0.32 to −0.22; p < 0.001; I^2^ = 88%). Non-survivor patients with COVID-19 had lower AGR values than survivor patients (MD, −0.29; 95% IC, −0.35 to −0.24; p < 0.001; I^2^ = 79%). In the sensitivity analysis, we only included studies with low risk of bias, which decreased the heterogeneity for both outcomes (severity, I^2^ = 20%; mortality, I^2^ = 5%).

**Conclusions:**

Low AGR values upon hospital admission were found in COVID-19 patients with a worse prognosis.

## 1 Introduction

At present, the coronavirus disease 2019 (COVID-19) pandemic, which is responsible for 4.5 million deaths at the time of writing [1], has been considered one of the most critical issues of global health. Due to the high number of COVID-19 cases in medical centers, tools are needed to help stratify patients and avoid the overloading and collapse of hospitals. In this context, the biomarkers are essential tools used when assessing a patient's prognosis with COVID-19 [2]. Several biomarkers that can predict the severity and risk of mortality in patients with SARS-COV-2 infection have been studied. Some examples of these are the neutrophil/lymphocyte ratio, the C-reactive protein, and the fibrinogen/albumin ratio, among others [3, 4, 5, 6, 7, 8].

The prognostic function of the albumin-to-globulin ratio (AGR) has been studied in several other pathologies, such as those of small cell lung cancer, hepatocellular carcinoma, heart failure, and infectious diseases [9, 10, 11, 12, 13]. In COVID-19 infection, the prognostic value of the AGR is based on the role that these serum proteins play in the process of systemic inflammation.

Increased globulin levels have been observed in patients with chronic inflammatory processes, such as those with hepatocellular carcinoma [14]. On the other hand, albumin levels have been shown to decrease in patients with altered nutritional status, systemic inflammation, and organ dysfunction [15, 16]. Moreover, serum albumin levels have been shown to be prognostic marker in COVID-19 patients [17, 18]. In this way, the AGR was postulated as a potential independent biomarker of both severity and mortality for COVID-19.

The aim of the present study is to evaluate and synthesize the published evidence regarding the ability of the AGR to predict the severity and mortality in patients diagnosed with SARS-COV-2 infection.

## 2 Methods

### 2.1 Report and register

The Preferred Reporting Items for Systematic Reviews and Meta-Analysis (PRISMA) [19] statement was used for reporting this systematic review and meta-analysis. Moreover, a short protocol version was registered in the International Prospective Register of Systematic Review (PROSPERO) [CRD42021275262].

### 2.2 Data sources and searches

Our search strategy was constructed in accordance with the Peer Review of Electronic Search Strategies (PRESS) Guidelines [20]. Studies evaluating the association between AGR and severity of COVID-19 patients were searched on September 20, 2021, in the following databases: PubMed, LILACS, Web of Science, Ovid MEDLINE, Embase, Scopus, and the WHO COVID-19 Global Research Database. Additionally, a manual search was performed in the CNKI databases, Wanfang Database, Research Square, medRixv, and SciELO Preprints (Table 1).

**Table 1:** Search strategy.

**PUBMED**
**Albumin (#1)** Albumin [MH] OR Albumins [MH] OR Serum Albumin [MH] OR Albumin, Serum [MH] OR Plasma Albumin [MH] OR Albumin [TIAB] OR Albumins [TIAB] OR “Serum Albumin” [TIAB] OR “Plasma Albumin” [TIAB] OR Albumin [OT] OR Albumins [OT] OR “Serum Albumin” [OT] OR “Plasma Albumin” [OT] **Globulin (#2)** Globulin [MH] OR Globulins [MH] OR Serum Globulins [MH] OR Globulins, Serum [MH] OR Pseudoglobulins [MH] OR Euglobulins [MH] OR Globulin [TIAB] OR Globulins [TIAB] OR “Serum Globulins” [TIAB] OR Pseudoglobulins [TIAB] OR Euglobulins [TIAB] OR Globulin [OT] OR Globulins [OT] OR “Serum Globulins” [OT] OR Pseudoglobulins [OT] OR Euglobulins [OT] **Albumin/Globulin ratio (#3)** “Albumin/globulin ratio” [OT] OR “Albumin/globulin index” [OT] OR “Ratio albumin/globulin” [OT] OR “Index albumin/globulin” [OT] OR “Albumin to globulin ratio” [OT] OR “Albumin-to globulin ratio” [OT] OR “Albumin to-globulin ratio” [OT] OR “Albumin-to-globulin ratio” [OT] OR “Albumin to globulin index” [OT] OR “Albumin-to globulin index” [OT] OR “Albumin to-globulin index” [OT] OR “Albumin-to-globulin index” [OT] OR “Ratio Albumin to globulin” [OT] OR “Ratio Albumin-to globulin” [OT] OR “Ratio Albumin to-globulin” [OT] OR “Ratio Albumin-to-globulin” [OT] OR “Index Albumin to globulin” [OT] OR “Index Albumin-to globulin” [OT] OR “Index Albumin to-globulin” [OT] OR “Index Albumin-to-globulin” [OT] **Covid-19 (#4)** COVID 19 [MH] OR COVID-19 Virus Disease [MH] OR COVID 19 Virus Disease [MH] OR COVID-19 Virus Diseases [MH] OR Disease, COVID-19 Virus [MH] OR Virus Disease, COVID-19 [MH] OR COVID-19 Virus Infection [MH] OR COVID 19 Virus Infection [MH] OR COVID-19 Virus Infections [MH] OR Infection, COVID-19 Virus [MH] OR Virus Infection, COVID-19 [MH] OR 2019-nCoV Infection [MH] OR 2019 nCoV Infection [MH] OR 2019-nCoV Infections [MH] OR Infection, 2019-nCoV [MH] OR Coronavirus Disease-19 [MH] OR Coronavirus Disease 19 [MH] OR 2019 Novel Coronavirus Disease [MH] OR 2019 Novel Coronavirus Infection [MH] OR 2019-nCoV Disease [MH] OR 2019 nCoV Disease [MH] OR 2019-nCoV Diseases [MH] OR Disease, 2019-nCoV [MH] OR COVID19 [MH] OR Coronavirus Disease 2019 [MH] OR Disease 2019, Coronavirus [MH] OR SARS Coronavirus 2 Infection [MH] OR SARS-CoV-2 Infection [MH] OR Infection, SARS-CoV-2 [MH] OR SARS CoV 2 Infection [MH] OR SARS-CoV-2 Infections [MH] OR COVID-19 Pandemic [MH] OR COVID 19 Pandemic [MH] OR COVID-19 Pandemics [MH] OR Pandemic, COVID-19 [MH] OR "COVID 19" [TIAB] OR "COVID-19 Virus Disease" [TIAB] OR "COVID 19 Virus Disease" [TIAB] OR "COVID-19 Virus Diseases" [TIAB] OR "COVID-19 Virus Infection" [TIAB] OR "COVID 19 Virus Infection" [TIAB] OR "COVID-19 Virus Infections" [TIAB] OR "Infection, COVID-19 Virus" [TIAB] OR "2019-nCoV Infection" [TIAB] OR "2019 nCoV Infection" [TIAB] OR "2019-nCoV Infections" [TIAB] OR "Coronavirus Disease-19" [TIAB] OR "Coronavirus Disease 19" [TIAB] OR "2019 Novel Coronavirus Disease" [TIAB] OR "2019 Novel Coronavirus Infection" [TIAB] OR "2019-nCoV Disease" [TIAB] OR "2019 nCoV Disease" [TIAB] OR "2019-nCoV Diseases" [TIAB] OR "COVID19" [TIAB] OR "Coronavirus Disease 2019" [TIAB] OR "Disease 2019, Coronavirus" [TIAB] OR "SARS Coronavirus 2 Infection" [TIAB] OR "SARS-CoV-2 Infection" [TIAB] OR "Infection, SARS-CoV-2" [TIAB] OR "SARS-CoV-2 Infections" [TIAB] OR "COVID-19 Pandemic" [TIAB] OR "COVID 19 Pandemic" [TIAB] OR "COVID-19 Pandemics" [TIAB] OR "COVID 19" [OT] OR "COVID-19 Virus Disease" [OT] OR "COVID 19 Virus Disease" [OT] OR "COVID-19 Virus Diseases" [OT] OR "COVID-19 Virus Infection" [OT] OR "COVID 19 Virus Infection" [OT] OR "COVID-19 Virus Infections" [OT] OR "Infection, COVID-19 Virus" [OT] OR "2019-nCoV Infection" [OT] OR "2019 nCoV Infection" [OT] OR "2019-nCoV Infections" [OT] OR "Coronavirus Disease-19" [OT] OR "Coronavirus Disease 19" [OT] OR "2019 Novel Coronavirus Disease" [OT] OR "2019 Novel Coronavirus Infection" [OT] OR "2019-nCoV Disease" [OT] OR "2019 nCoV Disease" [OT] OR "2019-nCoV Diseases" [OT] OR "COVID19" [OT] OR "Coronavirus Disease 2019" [OT] OR "Disease 2019, Coronavirus" [OT] OR "SARS Coronavirus 2 Infection" [OT] OR "SARS-CoV-2 Infection" [OT] OR "Infection, SARS-CoV-2" [OT] OR "SARS-CoV-2 Infections" [OT] OR "COVID-19 Pandemic" [OT] OR "COVID 19 Pandemic" [OT] OR "COVID-19 Pandemics" [OT] **Search Formula:** ((#1 AND #2) OR #3) AND #4
**WEB OF SCIENCE**
Query #1 Albumin OR Albumins OR Serum Albumin OR Albumin, Serum OR Plasma Albumin OR Albumin OR Albumins OR “Serum Albumin” OR “Plasma Albumin” OR Albumin OR Albumins OR “Serum Albumin” OR “Plasma Albumin” (All Fields) Query #2 ALL=(Globulin OR Globulins OR Serum Globulins OR Globulins, Serum OR Pseudoglobulins OR Euglobulins OR Globulin OR Globulins OR “Serum Globulins” OR Pseudoglobulins OR Euglobulins OR Globulin OR Globulins OR “Serum Globulins” OR Pseudoglobulins OR Euglobulins) Query #3 ALL=(“Albumin/globulin ratio” OR “Albumin/globulin index” OR “Ratio albumin/globulin” OR “Index albumin/globulin” OR “Albumin to globulin ratio” OR “Albumin-to globulin ratio” OR “Albumin to-globulin ratio” OR “Albumin-to-globulin ratio”OR “Albumin to globulin index” OR “Albumin-to globulin index” OR “Albumin to-globulin index” OR “Albumin-to-globulin index” OR “Ratio Albumin to globulin” OR “Ratio Albumin-to globulin” OR “Ratio Albumin to-globulin” OR “Ratio Albumin-to-globulin” OR “Index Albumin to globulin” OR “Index Albumin-to globulin” OR “Index Albumin to-globulin” OR “Index Albumin-to-globulin”) Query #4 ALL=(“COVID-19” OR “COVID 19” OR “COVID-19 Virus Disease” OR “COVID 19 Virus Disease” OR “COVID-19 Virus Diseases” OR “Disease, COVID-19 Virus” OR “Virus Disease, COVID-19” OR “COVID-19 Virus Infection” OR “COVID 19 Virus Infection” OR “COVID-19 Virus Infections” OR “Infection, COVID-19 Virus” OR “Virus Infection, COVID-19” OR “2019-nCoV Infection” OR “2019 nCoV Infection” OR “2019-nCoV Infections” OR “Infection, 2019-nCoV” OR “Coronavirus Disease-19” OR “Coronavirus Disease 19” OR “2019 Novel Coronavirus Disease” OR “2019 Novel Coronavirus Infection” OR “2019-nCoV Disease” OR “2019 nCoV Disease” OR “2019-nCoV Diseases” OR “Disease, 2019-nCoV” OR “COVID19” OR “Coronavirus Disease 2019” OR “Disease 2019, Coronavirus” OR “SARS Coronavirus 2 Infection” OR “SARS-CoV-2 Infection” OR “Infection, SARS-CoV-2” OR “SARS CoV 2 Infection” OR “SARS-CoV-2 Infections” OR “COVID-19 Pandemic” OR “COVID 19 Pandemic” OR “COVID-19 Pandemics” OR “Pandemic, COVID-19”) **Search Formula:** (((#1) AND #2) OR #3) AND #4
**OVIDMEDLINE**
1 ..nlpx "query=Albumin OR Albumins OR Serum Albumin OR Albumin, Serum OR Plasma Albumin OR Albumin OR Albumins OR “Serum Albumin” OR “Plasma Albumin” OR Albumin OR Albumins OR “Serum Albumin” OR “Plasma Albumin”","desiredResults=10000","minHitsDivisor=7","permitHyponyms=NO","lowestVocabularySearchLevel=none","phrasesBroken=NO","speedWanted=NoHypos","comment=Incluyendo términos relacionados","elimEnable=NO","constraintMinTerms=2" 2 limit 1 to full text 3 ..nlpx "query=Globulin OR Globulins OR Serum Globulins OR Globulins, Serum OR Pseudoglobulins OR Euglobulins OR Globulin OR Globulins OR “Serum Globulins” OR Pseudoglobulins OR Euglobulins OR Globulin OR Globulins OR “Serum Globulins” OR Pseudoglobulins OR Euglobulins","desiredResults=10000","minHitsDivisor=7","permitHyponyms=NO","lowestVocabularySearchLevel=none","phrasesBroken=NO","speedWanted=NoHypos","comment=Incluyendo términos relacionados","elimEnable=NO","constraintMinTerms=2" 4 limit 3 to full text 5 ..nlpx "query=“Albumin/globulin ratio” OR “Albumin/globulin index” OR “Ratio albumin/globulin” OR “Index albumin/globulin” OR “Albumin to globulin ratio” OR “Albumin-to globulin ratio” OR “v” OR “Albumin-to-globulin ratio”OR “Albumin to globulin index” OR “Albumin-to globulin index” OR “Albumin to-globulin index” OR “Albumin-to-globulin index” OR “Ratio Albumin to globulin” OR “Ratio Albumin-to globulin” OR “Ratio Albumin to-globulin” OR “Ratio Albumin-to-globulin” OR “Index Albumin to globulin” OR “Index Albumin-to globulin” OR “Index Albumin to-globulin” OR “Index Albumin-to-globulin” ","desiredResults=10000","minHitsDivisor=7","permitHyponyms=NO","lowestVocabularySearchLevel=none","phrasesBroken=NO","speedWanted=NoHypos","comment=Incluyendo términos relacionados","elimEnable=NO","constraintMinTerms=2" 6 limit 5 to full text 7 ..nlpx "query=“COVID-19” OR “COVID 19” OR “COVID-19 Virus Disease” OR “COVID 19 Virus Disease” OR “COVID-19 Virus Diseases” OR “Disease, COVID-19 Virus” OR “Virus Disease, COVID-19” OR “COVID-19 Virus Infection” OR “COVID 19 Virus Infection” OR “COVID-19 Virus Infections” OR “Infection, COVID-19 Virus” OR “Virus Infection, COVID-19” OR “2019-nCoV Infection” OR “2019 nCoV Infection” OR “2019-nCoV Infections” OR “Infection, 2019-nCoV” OR “Coronavirus Disease-19” OR “Coronavirus Disease 19” OR “2019 Novel Coronavirus Disease” OR “2019 Novel Coronavirus Infection” OR “2019-nCoV Disease” OR “2019 nCoV Disease” OR “2019-nCoV Diseases” OR “Disease, 2019-nCoV” OR “COVID19” OR “Coronavirus Disease 2019” OR “Disease 2019, Coronavirus” OR “SARS Coronavirus 2 Infection” OR “SARS-CoV-2 Infection” OR “Infection, SARS-CoV-2” OR “SARS CoV 2 Infection” OR “SARS-CoV-2 Infections” OR “COVID-19 Pandemic” OR “COVID 19 Pandemic” OR “COVID-19 Pandemics” OR “Pandemic, COVID-19”","desiredResults=10000","minHitsDivisor=7","permitHyponyms=NO","lowestVocabularySearchLevel=none","phrasesBroken=NO","speedWanted=NoHypos","comment=Incluyendo términos relacionados","elimEnable=NO","constraintMinTerms=2" 8 limit 7 to abstracts 9 2 and 4 10 6 or 9 11 8 and 10
**WHO’S GLOBAL RESEARCH ON CORONAVIRUS DISEASE (COVID-19) DATABASE**
tw:(Albumin/globulin ratio) AND collection:("01-internacional" OR "04-international_org" OR "09-preprints")
**SCOPUS**
(( ( ALL ( albumin OR albumins OR serum AND albumin OR albumin, AND serum OR plasma AND albumin OR albumin OR albumins OR "Serum Albumin" OR "Plasma Albumin" OR albumin OR albumins OR "Serum Albumin" OR "Plasma Albumin") ) AND (ALL ( globulin OR globulins OR serum AND globulins OR globulins, AND serum OR pseudoglobulins OR euglobulins OR globulin OR globulins OR "Serum Globulins" OR pseudoglobulins OR euglobulins OR globulin OR globulins OR "Serum Globulins" OR pseudoglobulins OR euglobulins) )) OR (ALL ( "Albumin/globulin ratio" OR "Albumin/globulin index" OR "Ratio albumin/globulin" OR "Index albumin/globulin" OR "Albumin to globulin ratio" OR "Albumin-to globulin ratio" OR "Albumin to-globulin ratio" OR "Albumin-to-globulin ratio" OR "Albumin to globulin index" OR "Albumin-to globulin index" OR "Albumin to-globulin index" OR "Albumin-to-globulin index" OR "Ratio Albumin to globulin" OR "Ratio Albumin-to globulin" OR "Ratio Albumin to-globulin" OR "Ratio Albumin-to-globulin" OR "Index Albumin to globulin" OR "Index Albumin-to globulin" OR "Index Albumin to-globulin" OR "Index Albumin-to-globulin") )) AND (TITLE-ABS-KEY ( "COVID-19" OR "COVID 19" OR "COVID-19 Virus Disease" OR "COVID 19 Virus Disease" OR "COVID-19 Virus Diseases" OR "Disease, COVID-19 Virus" OR "Virus Disease, COVID-19" OR "COVID-19 Virus Infection" OR "COVID 19 Virus Infection" OR "COVID-19 Virus Infections" OR "Infection, COVID-19 Virus" OR "Virus Infection, COVID-19" OR "2019-nCoV Infection" OR "2019 nCoV Infection" OR "2019-nCoV Infections" OR "Infection, 2019-nCoV" OR "Coronavirus Disease-19" OR "Coronavirus Disease 19" OR "2019 Novel Coronavirus Disease" OR "2019 Novel Coronavirus Infection" OR "2019-nCoV Disease" OR "2019 nCoV Disease" OR "2019-nCoV Diseases" OR "Disease, 2019-nCoV" OR "COVID19" OR "Coronavirus Disease 2019" OR "Disease 2019, Coronavirus" OR "SARS Coronavirus 2 Infection" OR "SARS-CoV-2 Infection" OR "Infection, SARS-CoV-2" OR "SARS CoV 2 Infection" OR "SARS-CoV-2 Infections" OR "COVID-19 Pandemic" OR "COVID 19 Pandemic" OR "COVID-19 Pandemics" OR "Pandemic, COVID-19") )
**LILACS**
Albumin [Palabras] and Globulin [Palabras] and Covid [Palabras]
**EMBASE**
Query Results #5 #3 AND #4 #4 #1 OR #2 #3 'covid-19':ab,ti OR 'covid 19':ab,ti OR 'covid-19 virus disease':ab,ti OR 'covid 19 virus disease':ab,ti OR 'covid-19 virus diseases':ab,ti OR 'disease, covid-19 virus':ab,ti OR 'virus disease, covid-19':ab,ti OR 'covid-19 virus infection':ab,ti OR 'covid 19 virus infection':ab,ti OR 'covid-19 virus infections':ab,ti OR 'infection, covid-19 virus':ab,ti OR 'virus infection, covid-19':ab,ti OR '2019-ncov infection':ab,ti OR '2019 ncov infection':ab,ti OR '2019-ncov infections':ab,ti OR 'infection, 2019-ncov':ab,ti OR 'coronavirus disease-19':ab,ti OR 'coronavirus disease 19':ab,ti OR '2019 novel coronavirus disease':ab,ti OR '2019 novel coronavirus infection':ab,ti OR '2019-ncov disease':ab,ti OR '2019 ncov disease':ab,ti OR '2019-ncov diseases':ab,ti OR 'disease, 2019-ncov':ab,ti OR 'covid19':ab,ti OR 'coronavirus disease 2019':ab,ti OR 'disease 2019, coronavirus':ab,ti OR 'sars coronavirus 2 infection':ab,ti OR 'sars-cov-2 infection':ab,ti OR 'infection, sars-cov-2':ab,ti OR 'sars cov 2 infection':ab,ti OR 'sars-cov-2 infections':ab,ti OR 'covid-19 pandemic':ab,ti OR 'covid 19 pandemic':ab,ti OR 'covid-19 pandemics':ab,ti OR 'pandemic, covid-19':ab,ti #2 'albumin/globulin ratio' OR 'albumin/globulin index' OR 'ratio albumin/globulin' OR 'index albumin/globulin' OR 'albumin to globulin ratio'/exp OR 'albumin to globulin ratio' OR 'albumin-to globulin ratio' OR 'albumin to-globulin ratio' OR 'albumin-to-globulin ratio' OR 'albumin to globulin index' OR 'albumin-to globulin index' OR 'albumin to-globulin index' OR 'albumin-to-globulin index' OR 'ratio albumin to globulin' OR 'ratio albumin-to globulin' OR 'ratio albumin to-globulin' OR 'ratio albumin-to-globulin' OR 'index albumin to globulin' OR 'index albumin-to globulin' OR 'index albumin to-globulin' OR 'index albumin-to-globulin' #1 ('albumin, serum':ab,ti OR albumin:ab,ti OR albumins:ab,ti OR 'serum albumin':ab,ti OR 'plasma albumin':ab,ti) AND ('globulins, serum':ab,ti OR globulin:ab,ti OR globulins:ab,ti OR 'serum globulins':ab,ti OR pseudoglobulins:ab,ti OR euglobulins:ab,t

### 2.3 Selection process and data extraction

All retrieved articles that complied with the following criteria were included: (i) case-control and cohort studies, (ii) studies on adult patients (≥18 years old) diagnosed with COVID-19, and (iii) studies assessing the relationship between the AGR values and severity or mortality in COVID-19 patients. Duplicates and studies that did not comply with the entirety of the eligibility criteria were excluded. COVID-19 severity was our primary outcome, defined as complying with at least one of these criteria: ICU admission, shortness of breath, respiration rate of ≥30 times per minute, blood oxygen saturation of ≤93% at rest, or PaO2/FiO2 ratio of ≤300 mmHg. However, definitions for severity vary widely among studies. Moreover, mortality was considered as a secondary outcome.

As for the study selection, four authors (JRUB, EAAB, MDMR, and EAHB) screened titles and abstracts independently using the data management software Rayyan QCRI (Rayyan Systems Inc©) [21]. Afterwards, the same four authors independently screened the remaining articles by full text against the eligibility criteria. Discrepancies were resolved through discussion until a consensus was reached. Finally, two authors (PHA and MDMR) extracted the data from selected studies in a standardized data extraction sheet in Microsoft Excel 2018 (Microsoft Corporation©). The extracted data included the following: first author, publication date, study title, study design, study location, population characteristics (number of participants, age, sex, comorbidities, and stratified sample data), exposures (mean or median AGR of the whole sample according to sample stratification) and outcomes types (severity or mortality).

### 2.4 Evaluation of study quality and publication bias

Two authors (EAAB, VB-Z) critically assessed the included studies using the Newcastle-Ottawa Scale (NOS) [22]. Risk of bias was categorized as low (scores ≥6), moderate (scores ranging 4–5), and high (scores <4). Moreover, funnel plots and Egger's tests were conducted to assess the publication bias. P-values of >0.1 were considered indicative of no publication bias.

### 2.5 Data synthesis and analysis

The effect size of each study was calculated using the means and standard deviations (SD) of the AGR values for severe vs. non-severe patients and survivor vs. non-survivor patients and pooled as mean difference (MD) with 95% confidence intervals (CI). Continuous data was presented as median and interquartile range (IQR) which was converted into relative means and SD by Hozo et al. [23]. The software Review Manager 5.4 (RevMan 5.4) (The Cochrane Collaboration, Copenhagen, Denmark) was used for statistical analysis. Statistical heterogeneity was determined using I^2^ statistics and categorized as severe (≥60%) and non-severe (<60%). Cochran's Q test was also conducted, and a p-value of <0.05 was considered statistically significant. A random-effects model was conducted as we anticipated heterogeneity among the studies. Subgroup analyses by study country (Chinese vs. non-Chinese studies) were performed, and the interaction between the p-value and each subgroup was investigated. Furthermore, we performed sensitivity analyses using studies with a low risk of bias.

## 3 Results

### 3.1 Study selection

**Figure 1:**
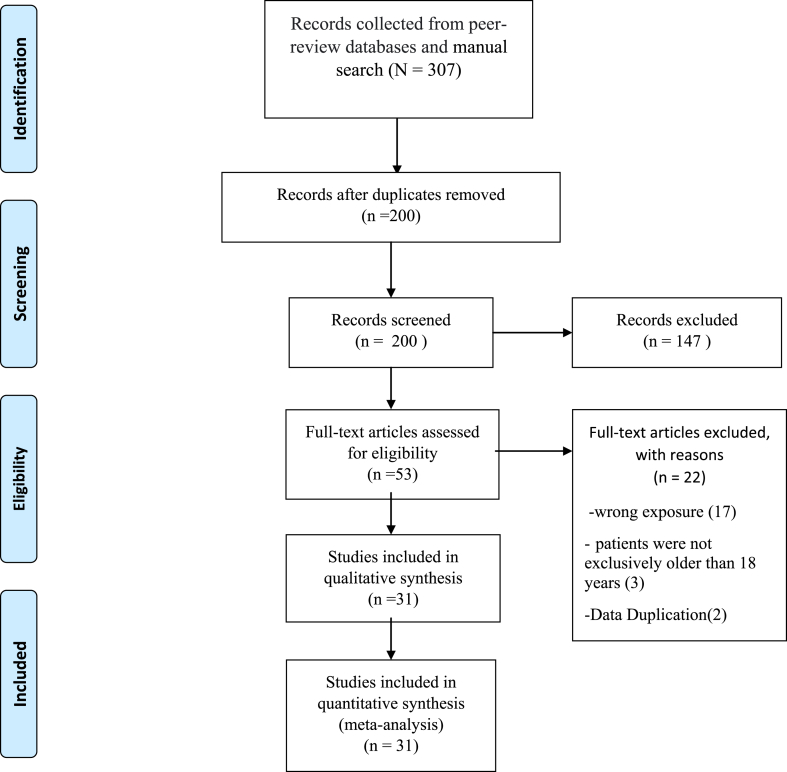
PRISMA Flow Diagram.

The systematic search retrieved 307 records, and after removing 107 duplicates, 200 records remained. Screening by titles and abstracts left 53 studies for full text review. A careful and rigorous assessment of full texts found 31 studies that complied with all eligibility criteria. This process is documented in a PRISMA flow diagram (Figure 1).

### 3.2 Study characteristics

All 31 included studies were cohort studies, and their characteristics are outlined in Table 2 and Table 3 [24, 25, 26, 27, 28, 29, 30, 31, 32, 33, 34, 35, 36, 37, 38, 39, 40, 41, 42, 43, 44, 45, 46, 47, 48, 49, 50, 51, 52, 53, 54]. Six studies compared the AGR values upon hospital admission of survival and non-survival COVID-19 patients, and the remaining 25 studies compared the AGR values upon hospital admission between severe and non-severe COVID-19 patients. Moreover, 4 studies were conducted in India, 1 in Turkey, 1 in Algeria, 1 in Japan, and the remaining 24 in China.

**Table 2:** Characteristics of the included studies comparing severe and non-severe COVID-19 patients.

Author	Year	Country	Participants (male)	Median/mean age (IQR/SD)	Comorbidities (n)			AGR mean in severe patients	AGR mean in non-severe patients	Cut-off
					Diabetes mellitus	Hypertension	Obesity			
Fu L et al*.*	2020	China	350 (190)	NR	145	125	NR	1.37 (0.37)	1.37 (0.22)	NR
Guiling L et al.	2020	China	107 (53)	65 (60–70)	12	44	NR	1.32 (0.22)	1.47 (0.22)	NR
Gemcioglu E et al.	2021	Turkey	301 (161)	45 (24)	107	169	4	1.46 (0.59)	1.87 (0.39)	1.52
Cao Z et al.	2020	China	80 (38)	53 (20)	6	20	NR	0.8 (0.2)	1.1 (0.2)	NR
Shi S et al*.*	2021	China	87 (49)	60 (22–88)	NR	NR	NR	0.91 (0.25)	1.24 (0.23)	1
Yang R et al*.*	2020	China	495 (235)	55 (40–67)	47	137	NR	1.21 (0.24)	1.50 (0.31)	NR
Tsui E et al.	2020	China	535 (264)	37.8 (18)	33	87	NR	1.01 (0.30)	1.33 (0.30)	NR
Bennouar S et al*.*	2020	Algeria	330 (206)	66.6 (9)	NR	NR	NR	1.0 (0.18)	1.43 (0.3)	1.15
Huang Jiana et al*.*	2021	China	98 (46)	44 (33–62)	7	19	NR	1.23 (0.22)	1.39 (0.24)	NR
Mishra C et al*.*	2021	India	500 (308)	60 (16.3)	126	259	NR	0.94 (0.23)	1.16 (0.3)	NR
Wang Changzheng et al*.*	2020	China	45 (23)	39 (18–62)	4	4	NR	1.23 (0.23)	1.71 (4.02)	NR
Fang Z et al*.*	2020	China	239 (118)	54.87 (14)	14	28	NR	1.32 (0.28)	1.54 (0.3)	NR
Wang Menghan et al*.*	2021	China	151 (64)	63 (14)	32	60	NR	0.96 (6.84)	1.32 (0.29)	NR
Zhao C et al*.*	2021	China	172 (82)	65 (57–71)	27	63	NR	1.09 (0.28)	1.42 (0.37)	NR
Kalal CR et al*.*	2021	India	134 (89)	45.5 (18–86)	31	40	NR	1.07 (0.22)	1.4 (0.29)	NR
Huang Juan et al*.*	2020	China	1187 (537)	55 (32–76)	NR	309	NR	1.36 (0.33)	1.52 (0.37)	NR
Shang H et al*.*	2020	China	514 (271)	54 (48–68)	99	222	NR	0.9 (0.29)	1.02 (0.22)	NR
Chen Xu et al*.*	2020	China	291 (145)	46 (34–59)	22	39	NR	1.19 (0.3)	1.33 (0.52)	1.5
Yamamoto A et al*.*	2021	Japon	152 (74)	53.5 (38–70)	11	17	NR	1.02 (0.14)	1.42 (0.22)	1.1
Barya P et al*.*	2021	India	75 (43)	47.51 (20–90)	16	4	NR	0.89 (0.28)	1.43 (0.28)	1.1
Fang Hu et al*.*	2020	China	91 (40)	47.53 (15)	9	17	NR	1.24 (0.18)	1.38 (0.22)	NR
Qi J et. al	2020	China	104 (47)	42 (33–56)	NR	NR	NR	1.1 (0.14)	1.32 (0.24)	1.2
Xu F et al*.*	2020	China	251 (132)	60 (16)	NR	NR	NR	0.82 (0.22)	1.1 (0.29)	NR
Dai Wanfa et al*.*	2020	China	61 (40)	50 (17)	6	16	NR	1.12 (0.23)	1.43 (0.51)	NR
Bing H et al*.*	2020	China	53 (28)	50 (27–68)	NR	NR	NR	1.1 (0.29)	1.75 (0.44)	NR

NR: NOT REPORTED.

**Table 3:** Characteristics of the included studies comparing survivor and non-survivor COVID-19 patients.

Author	Year	Country	Participants (male)	Median/mean age (IQR/SD)	Comorbidities (n)			AGR mean in non-survivor patients	AGR mean in survivor patients	Cut-off
					Diabetes Mellitus	Hypertension	Obesity			
Wei Y et al*.*	2020	China	112 (73)	61 (14.9)	21	40	NR	1.2 (0.2)	1.5 (0.3)	NR
Wang Xue et al*.*	2020	China	131 (56)	64 (56–71)	28	52	NR	0.9 (0.17)	1.27 (0.25)	NR
Caillon A et al*.*	2021	China	157 (75)	64 (46–76)	24	55	NR	1.09 (0.22)	1.34 (0.26)	NR
Wang Kun et al*.*	2020	China	296 (140)	47.32 (14.95)	30	42	NR	1.2 (0.3)	1.6 (0.4)	NR
Elavarasi A et al*.*	2021	India	2017 (1320)	47.4 (17.6)	437	457	NR	1.3 (0.29)	1.52 (0.22)	NR
Huang Wei et al*.*	2020	China	2240 (1136)	64 (52–71)	NR	NR	NR	0.85 (0.17)	1.16 (0.31)	NR

NR: NOT REPORTED.

A total of 11356 patients were evaluated, of which 53.56% were men. In 15 studies, the days elapsed for the development of severity from the day of admission were reported, ranging between 5 and 17 days. Only one study did not report the age of the patients. Regarding the comorbidities of the included patients, a total of 1294 (11.4%) diabetes patients, 2325 (20.47%) hypertensive patients, and 4 (0.03%) overweight patients were recorded.

Seven studies evaluated the optimal AGR cut-off values for severity that ranged from 1 to 1.52. Likewise, no studies have evaluated the AGR cut-off values for mortality. Fourteen studies had low risk of bias in the study quality appraisal, while the remaining 17 had a moderate to high risk of bias (Table 4).

**Table 4:** Newcastle - Ottawa quality assessment scale for included studies.

Newcastle - Ottawa Quality Assessment Scale for Cohort Studies										
Study	Selection				Comparability	Outcome			Score	Evidence quality
	Representativeness of the exposed cohort	Selection of the non-exposed cohort	Ascertainment of exposure	Demonstration that outcome of interest was not present at start of study	Comparability of Cohorts on the Basis of the Design or Analysis Maximum: ☆☆	Assessment of outcome	Was follow-up long enough for outcomes to occur	Adequacy of follow up of cohorts		
Fu L et al.	☆	☆	☆			☆			*4*	Moderate risk of bias
Guiling L et al.		☆	☆			☆	☆	☆	*5*	Moderate risk of bias
Gemcioglu E et al.	☆	☆	☆			☆	☆	☆	*6*	Low Risk of bias
Cao Z et al.		☆	☆	☆	☆	☆	☆	☆	*7*	Low Risk of bias
Shi S et al.	☆	☆	☆			☆	☆	☆	*6*	Low Risk of bias
Yang R et al.		☆	☆	☆		☆	☆	☆	*6*	Low Risk of bias
Tsui E et al.	☆	☆	☆			☆	☆	☆	*6*	Low Risk of bias
Huang Jiana et al.		☆	☆	☆		☆	☆	☆	*6*	Low Risk of bias
Mishra C et al.		☆	☆			☆	☆	☆	*5*	Moderate risk of bias
Wang Changzheng et al.		☆	☆			☆	☆		*4*	Moderate risk of bias
Fang Z et al.		☆	☆			☆		☆	*4*	Moderate risk of bias
Wang Menghan et al.			☆			☆		☆	*3*	High Risk of bias
Zhao C et al.	☆	☆	☆		☆	☆	☆	☆	*7*	Low Risk of bias
Kalal CR et al.		☆	☆		☆	☆	☆	☆	*6*	Low Risk of bias
Huang Juan et al.		☆	☆			☆		☆	*4*	Moderate risk of bias
Shang H et al.		☆	☆	☆		☆			*4*	Moderate risk of bias
Chen Xu et al.		☆	☆		☆	☆	☆		*5*	Moderate risk of bias
Yamamoto A et al.		☆	☆		☆	☆	☆		*5*	Moderate risk of bias
Barya P et al.		☆	☆			☆			*3*	High Risk of bias
Fang Hu et al.				*☆*	*☆*	*☆*		*☆*	*4*	Moderate risk of bias
Qi J et al.			*☆*	*☆*	*☆*	*☆*	*☆*	*☆*	*6*	Low Risk of bias
Xu F et al.			*☆*	*☆*	*☆*	*☆*	*☆*	*☆*	*6*	Low Risk of bias
Wei Y et al.		*☆*	*☆*	*☆*	*☆*		*☆*	*☆*	*6*	Low Risk of bias
Wang Xue et al.	*☆*	*☆*	*☆*	*☆*	*☆*	*☆*	*☆*	*☆*	*8*	Low Risk of bias
Caillon A et al.		*☆*	*☆*	*☆*	*☆*	*☆*	*☆*	*☆*	*7*	Low Risk of bias
Wang Kun et al.			*☆*	*☆*	*☆*	*☆*			*4*	Moderate risk of bias
Elavarasi A et al.			*☆*	*☆*		*☆*	*☆*		*4*	Moderate risk of bias
Huang Wei et al.	*☆*	*☆*	*☆*	*☆*	*☆*	*☆*	*☆*	*☆*	*8*	Low Risk of bias
Dai Wanfa et al.				*☆*	*☆*	*☆*		*☆*	*4*	Moderate risk of bias
Bing H et al.		☆	☆		☆	☆	☆		*5*	Moderate risk of bias

### 3.3 AGR and COVID-19 severity

**Figure 2:**
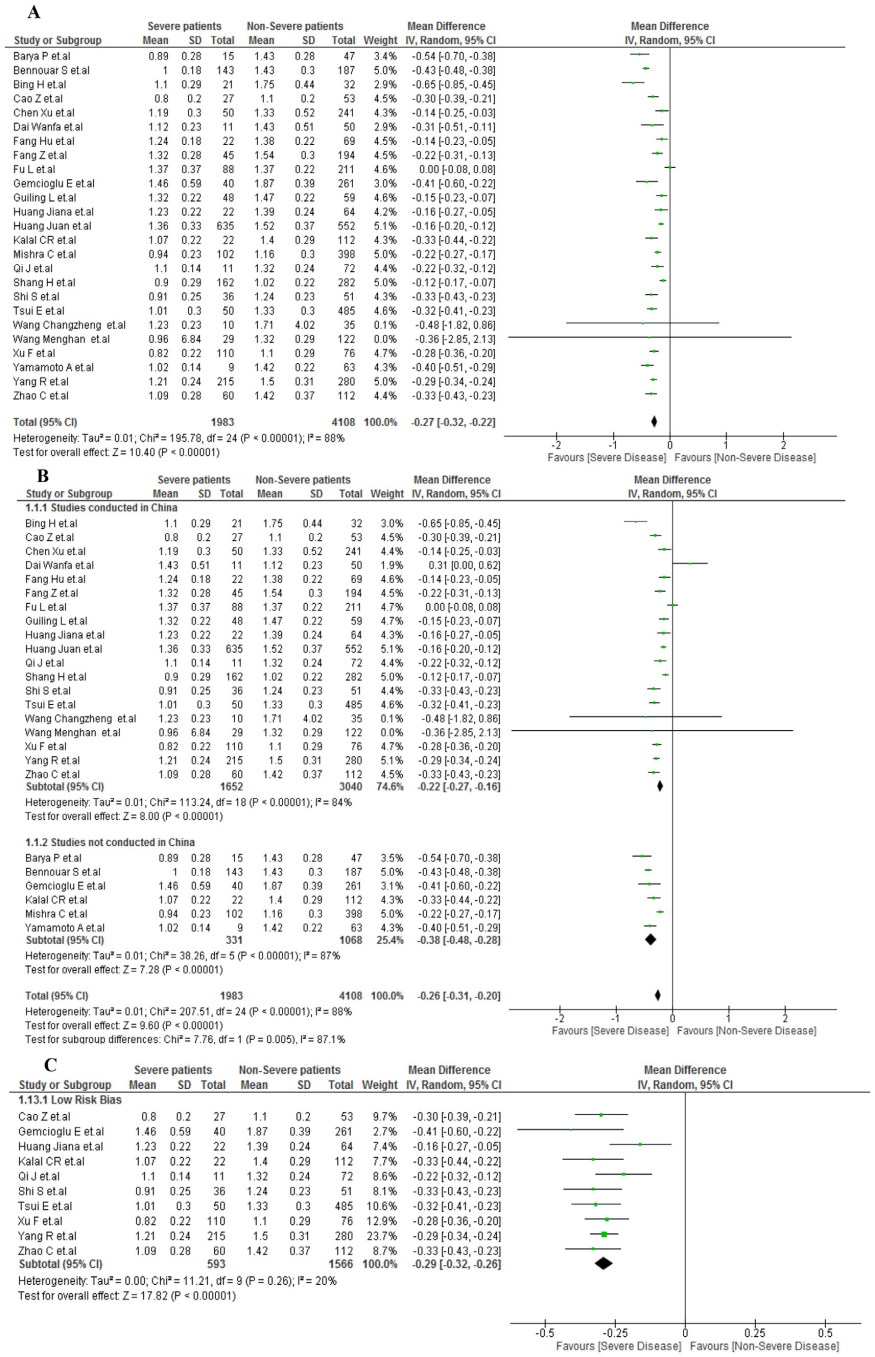
A. AGR values in severe vs non-severe COVID-19 patients. B. Subgroup analysis according to country of origin between severe vs nonsevere COVID-19 patients. C. Sensitivity analysis according to the risk of bias between severe vs nonsevere COVID-19 patients.

A total of 6081 patients were evaluated in 25 included articles, with 1983 patients developing severe COVID-19. Patients with severe COVID-19 had lower AGR values than non-severe COVID-19 patients (MD, −0.27; 95% IC, −0.32 to −0.22; p < 0.001) (Figure 2A). However, due to severe heterogeneity (I^2^ = 88%), a subgroup analysis was performed by study location. No significant differences were observed between the Chinese studies (MD, −0.22; 95% CI, −0.27 to −0.16; p < 0.001) and non-Chinese studies (MD, −0.38; 95% CI, −0.48 to −0,28; p < 0,001), with an interaction test p-value of <0.002 and severe heterogeneity in both subgroups (I^2^ > 85%) (Figure 2B). The sensitivity analysis included only low risk of bias studies and showed the same result: low AGR values for severe COVID-19 patients (MD, −0.29; 95% CI, −0.32 to −0.26; p < 0.001). Nonetheless, heterogeneity decreased significantly (I^2^ = 20%) (Figure 2C).

### 3.4 AGR and COVID-19 mortality

**Figure 3:**
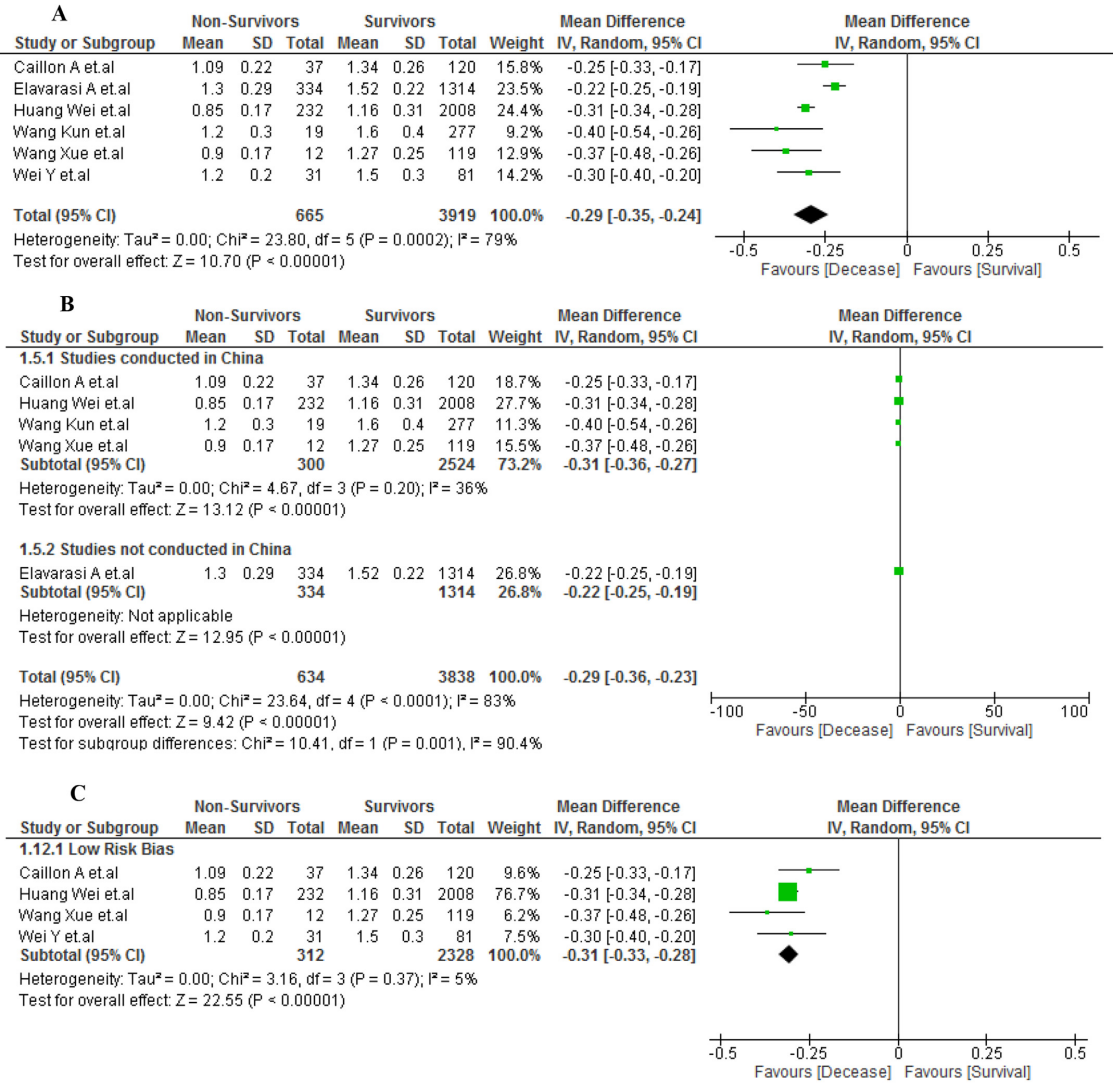
A. AGR values in survivors vs non-survivors COVID-19 patients. B. Subgroup analysis according to country of origin between survivors vs non-survivors COVID-19 patients. C. Sensitivity analysis according to the risk of bias between survivors vs non-survivors COVID-19 patients.

A total of 4584 patients were evaluated in six included articles, of which 665 patients diagnosed with COVID-19 died after hospitalization. The non-survivor patients with COVID-19 had lower AGR values than survivor patients (MD, −0.29; 95% IC, −0.35 to −0.24; p < 0.001) (Figure 3A). However, due to severe heterogeneity (I^2^ = 79%), a subgroup analysis was performed by study location. A mild heterogeneity (I^2^ = 36%) was obtained from the Chinese studies (MD, −0.31; 95% CI, −0.36 to −0.27; p < 0.001) (Figure 3B). The sensitivity analysis included only low risk of bias studies and showed the same result: low AGR values for non-survivor patients of COVID-19 (MD, −0.31; 95% CI, −0.33 to −0.28; p < 0.001). However, heterogeneity decreased significantly (I^2^ = 5%) (Figure 3C).

### 3.5 Publication bias

**Figure 4:**
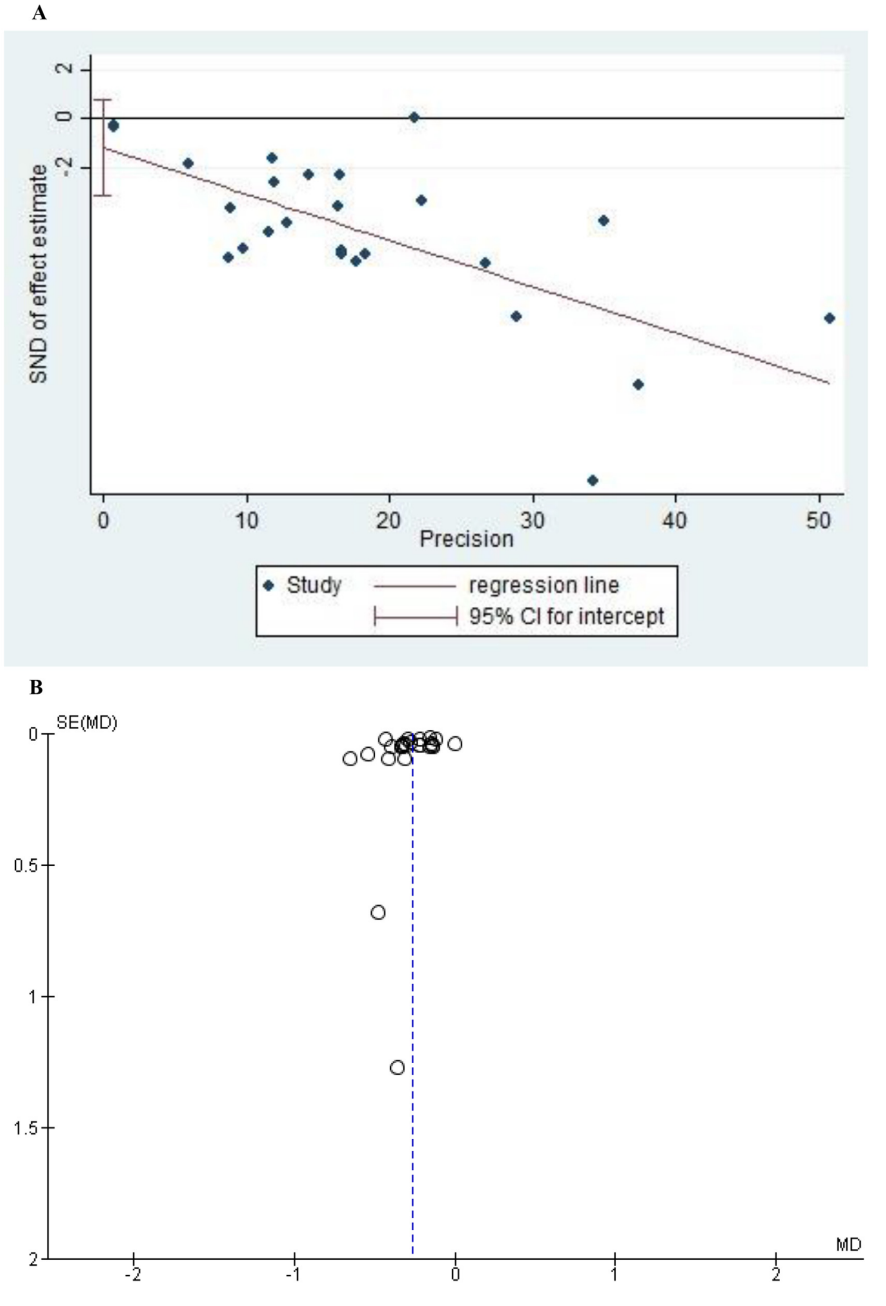
A: Egger Test of all the studies that evaluated AGR values in severe vs non-severe COVID-19 patients. B: Funnel Plot of the studies that evaluated AGR values in severe vs nonsevere COVID-19 patients.

**Figure 5:**
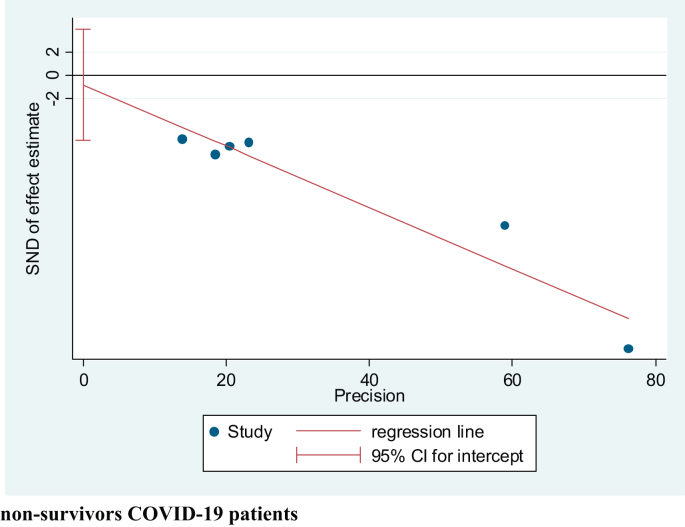
Egger Test of all the studies that evaluated AGR values in survivors vs non-survivors COVID-19 patients.

The Egger's tests performed to evaluate the publication bias of the studies (Figures 4A and 5) assessing the mortality and severity of the disease showed no indication of small-study effects (p = 0.658 and p = 0.219, respectively). Moreover, the funnel plot for the studies assessing severity showed a symmetrical pattern (Figure 4B).

## 4 Discussion

The present systematic review and meta-analysis found that upon hospital admission, low AGR values were observed in COVID-19 patients who developed severe disease or died compared with patients who survived or did not develop severe disease.

Albumin and globulin are two primary components of serum proteins and are involved in systemic inflammation. Low albumin levels are associated with malnutrition and inflammation, whereas low globulin levels are associated with chronic inflammation and indicate cumulative exposure to various pro-inflammatory cytokines [55]. AGR is considered a more stable and reliable marker than serum albumin or globulin alone as a prognostic factor and has been associated with poor results in relation to different pathologies, such as digestive and urological cancers and solid cancers in general [56, 57, 58]. Similarly, it was associated with the prognosis of other diseases, such as heart failure and chronic kidney disease [10, 59]. In infectious diseases, AGR was associated with a worse prognosis in pediatric patients with tuberculous meningitis as a predictor of febrile urinary tract infection after ureteroscopic lithotripsy [60, 61].

In patients with COVID-19, the role of inflammation as a marker of severity and poor prognosis is suggested. A systematic review and meta-analysis of 23 studies found that in patients with severe COVID-19, high values of procalcitonin, C-reactive protein, D-dimer, and lactic dehydrogenase were found, as well as low values of albumin [62]. Similarly, a meta-analysis of 17 articles found a marked decrease in the values of lymphocytes, monocytes, eosinophils, platelets, albumin, the C-reactive protein/lymphocyte ratio, and the C-reactive protein/leukocyte ratio [63]. Likewise, the authors found high values of C-reactive protein, glomerular sedimentation rate, procalcitonin, lactate dehydrogenase, and others [63]. Thus, the inflammation in patients with COVID-19 raised the need to identify more stable markers to predict prognosis. In this way, the C-reactive protein/lymphocyte ratio, C-reactive protein/leukocyte ratio, neutrophil/monocyte ratio, and neutrophil/lymphocyte ratio demonstrated their potential as prognostic biomarkers in patients with sepsis [64, 65].

As mentioned, AGR is a better and more stable prognostic marker than albumin and globulin alone. Therefore, its association with severity and mortality in patients with COVID-19 may be related to the properties of these proteins. A meta-analysis of 67 studies had found that low albumin levels were associated with severity and worse prognosis in patients with COVID-19 [66]. Although the reasons for this association are not clear, its relationship to inflammation is believed to be one of them. A low level of albumin is related to the release of cytokines, including interleukin (IL)-6 and tumor necrosis factor (TNF)-α, and is inversely related to other markers, such as the neutrophil/lymphocyte ratio [57]. Therefore, a lower AGR value indicates more inflammation, which predicts more severity of the disease and, therefore, a greater probability of dying. Likewise, it is likely that as albumin values are influenced by other parameters independently associated with the severity and poorer prognosis of patients with COVID-19, they contribute to the prognostic value of the AGR, for example, age. In line with this, various meta-analyses had shown the association of older age with severity and poor prognosis in these patients [67]. On the other hand, various inflammation markers are associated with other sociodemographic factors of the AGR that were not evaluated and associated with worse prognosis in COVID-19 patients, which could confirm the findings of this study [68].

Although systematic reviews and meta-analyses on the role of various inflammation markers and their prognostic value in patients with COVID-19 have been published [4, 60], to our knowledge, this is the first study that focuses on the AGR in relation to COVID-19. Similarly, we performed a sensitivity analysis taking into account the biases of the studies, which gives robustness to our results. Likewise, our findings will allow us to suggest a potential low-cost prognostic marker in patients hospitalized for COVID-19 that will encourage health personnel to prioritize or individualize management strategies in patients with low AGR values.

### 4.1 Limitations

Our study needs to be rationally interpreted due to its limitations. First, the meta-analyses of both outcomes presented high heterogeneity, which indicates the clinical and methodological differences of the analyzed studies. However, we could explain this heterogeneity, which was led by studies that were not at low risk of bias and by differences between countries. Second, the calculated estimates come from averages without adjusting for confounding variables. Furthermore, it is necessary to conduct more epidemiological studies where an optimal cut-off point for the AGR and its prognostic value adjusted by confounding factors is defined. Third, the small sample size of some studies could cause them to be over-represented in the meta-analysis. However, the results of Egger's test did not show any statistical significance for the small-study effect. Fourth, although no language or region restriction was applied in the systematic search, most of the included studies have been published in Asia, so the assessment of other populations should be addressed in future research. Fifth, same levels of physiological parameters across countries are not directly comparable and possible levels of albumin and globulin need to have a geographical context when we say high or low. Sixth, the included studies did not consider the modulatory role played by factors, such as age, gender, and the presence or absence of comorbidities. Likewise, it is also critical to have the context of the samples included in the different studies, since it is possible that the potential role of the virus in modulating the levels of albumin and globulin has evolved. Finally, we cannot confirm the role of this marker in non-severe patients, and although the role of inflammation was suggested, the studies did not consider these markers to confirm this hypothesis.

## 5 Conclusion

The AGR is a biomarker that can predict the severity and mortality of COVID-19 patients. Moreover, low AGR values upon hospital admission were observed in COVID-19 patients who developed severe disease or died.

## Declarations

### Author contribution statement

Juan R. Ulloque-Badaracco, Melany D. Mosquera-Rojas, Enrique A Hernandez-Bustamante, Esteban A Alarcón-Braga: Conceived and designed the experiments; Performed the experiments; Analyzed and interpreted the data; Wrote the paper.

Percy Herrera – Añazco and Vicente A. Benites-Zapata: Contributed reagents, materials, analysis tools or data; Wrote the paper.

### Funding statement

This research did not receive any specific grant from funding agencies in the public, commercial, or not-for-profit sectors.

### Data availability statement

Data included in article/supplementary material/referenced in article.

### Declaration of interests statement

The authors declare no conflict of interest.

### Additional information

No additional information is available for this paper.
